# Supplementary material for: Power-free knee rehabilitation robot for home-based isokinetic training
Source: Nat Commun. 2025 Mar 8;16:2347. doi: 10.1038/s41467-025-57578-z (PMC11890594; doi:10.1038/s41467-025-57578-z)
Supplement: Supplementary file 1 — Supplementary Information [file 41467_2025_57578_MOESM1_ESM.pdf]

Supplementary Information

This file includes:

Supplementary text

Supplementary Figures 1 to 9

Supplementary Table 1 to 10

Supplementary Text

Mechanical Assembly

The detailed mechanical assembly is shown in Supplementary Fig. S1. The steel structure frame and the complete mechanical structure are divided into the human-machine interface in Supplementary Fig. S1a and the actuator in Supplementary Fig. S1b. Besides the main steel support frame structure and other accompanying components, e.g., chest and leg straps and smartphone holder to fix the smartphone for human-machine interface. During the training, the control circuit sends training data, e.g., angle, torque, and speed to the smartphone via Bluetooth, and the peak torque of each repetition, including lifting and retracting, is displayed on the smartphone as a bar plot. The bar charts of lifting and retracting are differentiated using different colors. The average value of the entire training session is displayed at the bottom of the interface when the subject completes all repetitions in one exercise. After each exercise, the complete training data will be sent to the cloud, enabling the professional physician to provide treatment recommendations. The actuator includes motor assembly, control circuit, and fixture assembly (see Supplementary Fig. S1b). The motor assembly includes the main arm, torque sensor, adapter sleeve and adapter plate, BLDC motor and angle sensor. The control circuit is for controlling, driving, data transmitting, and most importantly, energy regeneration.

Detailed Bio-mechanical Energy Production Model

The knee joint is regarded as a bio-mechanical system comprising two skeleton bones, the femur and tibia, as well as two antagonistic muscles, quadriceps femoris (dominating leg lifting) and hamstring muscle (dominating leg retracting), as shown in Supplementary Fig. S6. The angle between the femur and tibia denotes the knee angle θ, and $\dot{\theta}$ represents the angular velocity of knee flexion, and F_Q is the contractile force of the quadriceps femoris, and F_H is the force of the hamstring muscle and G is the gravity of the calf. The moment arms of the three forces are l_Q, l_H and l_G, respectively. T is an overall torque from the knee joint, which is exerted on the isokinetic training robot.

Based on the balance condition of the moment, the torque T is equal to the sum of the contractile torques of the quadriceps femoris (T_Q), hamstring muscle (T_H) and torques generated by the weight of calf (T_G), $T_H = F_H \cdot l_H$, $T_Q = F_Q \cdot l_Q$, $T_G = G \cdot l_G$:

$$T = T_H + T_Q + T_G \qquad (S1)$$

The muscle (quadriceps femoris) can be regarded as a combination of serial element (SE), contractile element (CE), and parallel element (PE) according to Hill's 3-elements muscle theory (*46*), as shown in Supplementary Fig. S6. The total contractile force of the quadriceps femoris is equal to the force of SE (F_{SE}), which is the summation of the contractile force of CE (F_{CE}) and the passive force of PE (F_{PE}), i.e., $F = F_{CE} + F_{PE}$. F_{CE} can be described as a function involving four factors (*47*), i.e., the maximum voluntary contractile force (MVC, denoted by F_m), the activation level (α), the force-length factor (w_l) and the force-velocity factor (w_v). F_{PE} can be considered negligible over most of the motion range and with a peak of less than 10% of F_m at the maximum anatomical length (*48*), i.e., F_{PE} = 0, thus F_q is equal to the following equation

$$F_Q \approx F_{CE} = F_m \cdot \alpha \cdot w_l + w_v \qquad (S2)$$

During leg lifting, the hamstring muscle is assumed not to contribute to co-contractions in maximum flexions, i.e., $T_H = 0$. After the self-weight calibration by the isokinetic training robot, i.e., $T_G = 0$. Thus, the torque T is contributed by T_Q exclusively

$$T \approx T_Q = F_Q \cdot l_Q \approx F_{CE} \cdot l_Q \quad (S3)$$

When maximum contractile torque T_m is generated by the quadriceps femoris, F_{op} is the contractile force, and l_{op} is the moment arm, i.e., $T_m = F_{op} \cdot l_{op}$. The elbow joint and knee joint are similar in physical structure and function, and thus it is reasonable and natural to consider extending the elbow system model to the knee joint. In the knee muscular-skeleton system, the generated torque in extensions also depends on the muscle activation level α, the knee angle θ, and angular velocity $\dot{\theta}$ (*47*), as shown in the following equation

$$T \approx T_m \cdot \alpha \cdot \widetilde{w_l}(\theta) \cdot w_v(\dot{\theta}) \quad (S4)$$

$$\widetilde{w_l} = \frac{F_m}{F_{op}} \cdot \frac{l_Q(\theta)}{l_{op}} \cdot w_l(\theta) \quad (S5)$$

The muscle activation level α can be described as a function of the circumferential strain s for isometric flexions according to the following studies (*34-36*). An approximation of activation level is assumed as follows, where $b = 0$, k_1 and k_2 are functions of knee angle and angular velocity, respectively (*49*)

$$\alpha = \alpha(s) \approx ks + b \quad (S6)$$

$$k = k_1(\theta) \cdot k_2(\dot{\theta}) \quad (S7)$$

Substitute Eq. S6 and Eq. S7 to Eq. S4

$$T \approx T_m \cdot s \cdot W_l(\theta) \cdot W_v(\dot{\theta}) \quad (S8)$$

$$W_l = k_1(\theta) \cdot \widetilde{w_l}(\theta) \quad (S9)$$

$$W_v = k_2(\dot{\theta}) \cdot w_v(\dot{\theta}) \quad (S10)$$

$s_{max}(\theta)$ is the maximum circumferential strain obtained in isometric MVC flexions at knee angle θ, and β ($0 \le \beta \le 1$) is the normalized circumferential strain, which is the ratio of s to $s_{max}(\theta)$ (*49*). The final knee joint torque model is arranged as

$$T \approx T_m \cdot \beta \cdot \widetilde{W_l}(\theta) \cdot \widetilde{W_v}(\dot{\theta}) \quad (S11)$$

$$\widetilde{G_l} = k_1(\theta) \cdot s_{max}(\theta) \cdot \frac{F_m}{F_{op}} \cdot \frac{l_Q(\theta)}{l_{op}} \cdot w_l(\theta) \quad (S12)$$

$$\widetilde{W_v} = k_2(\dot{\theta}) \cdot w_v(\dot{\theta}) \tag{S13}$$

T is an overall torque from the knee joint, which is exerted on the isokinetic training robot. More details can refer to Eq. S1-S13. According to the induction electromotive force formula of the DC motor, *T* is the drag torque, C_r is the motor torque constant, *Φ* is the air gap flux of each magnetic pole, phase current I_a can be expressed as Eq. S14 at a certain time, i.e., the relationship between muscle contraction and electric energy regeneration has been established,

$$I_a = \frac{T}{C_r \Phi} \tag{S14}$$

Detailed Charging Principle

During the training, the energy was harvested to the ultracapacitor by the control circuit. Subsequent sections provide detailed information on the charging principles within the control circuit. According to Kirchhoff's Voltage Law, the corresponding differential equation while MOSFETs *S2*, *S4,* and *S6* ON, as shown in Supplementary Fig. S3, can be expressed as

$$E_{b_{on}} - E_{a_{on}} = (R_a + R_{s2})I_{a_{on}} + (R_b + R_{s4})I_{b_{on}} + L_a \frac{dI_{a_{on}}}{dt_{a_{on}}} + L_b \frac{dI_{b_{on}}}{dt_{a_{on}}} \tag{S15}$$

where R_{s2} and R_{s4} represent the equivalent resistance of MOSFETs *S2* and *S4*, respectively, and $t_{a_{on}}$ indicates the increasing time of the current $I_{a_{on}}$.

When the MOSFETs *S2*, *S4*, and *S6* are switched OFF, the back EMFs and currents on *a* and *b* are expressed as $E_{a_{off}}$, $E_{b_{off}}$, $I_{a_{off}}$ and $I_{b_{off}}$ respectively. According to Kirchhoff's Voltage Law, the corresponding differential equation while MOSFETs *S2*, *S4*, and *S6* OFF, as shown in Supplementary Fig. S7, can be expressed as

$$E_{b_{off}} - E_{a_{off}} = R_a I_{a_{off}} + R_b I_{b_{off}} + U_{uc} + U_{FD1} + U_{FD4} + L_a \frac{dI_{a_{off}}}{dt_{a_{off}}} + L_b \frac{dI_{b_{off}}}{dt_{a_{off}}} \tag{S16}$$

$$C \frac{dU_{uc}}{dt} = I_{a_{off}} \tag{S17}$$

where U_{FD1} and U_{FD4} refer to the diode forward conduction voltages of *D1* and *D4* respectively, and U_{uc} and *C* represent the voltage and capacitance of the ultracapacitor, respectively, and $t_{a_{off}}$ indicates the decreasing time of the current $I_{a_{off}}$.

Ignoring the individual effects of components (i.e., $R_{s1} = R_{s2} = R_s$, U_{FD1}=U_{FD4}= U_{FD}, I_a = I_b), the increasing current $dI_{a_{on}}$ through the equivalent inductor L_a and the decreasing current $dI_{a_{off}}$ through the equivalent inductor L_a in one cycle can be respectively expressed as

$$dI_{a_{on}} = \frac{\left(E_b - E_a - 2(R_a + R_s)\overline{I_a}\right)DT_s}{2L_a} \tag{S18}$$

$$dI_{a_{off}} = \frac{\left(\overline{U_{uc}} + 2U_{FD} + 2R_a\overline{I_a} - (E_b - E_a)\right)\Delta T_s}{2L_a} \quad (S19)$$

where T_s refers to the duration of a cycle and $\overline{U_{uc}}$ refers to the average voltage of the ultracapacitor. $\overline{I_a}$ represents the average current flowing through the equivalent inductor L_a in one cycle, *D* is the duty cycle of the PWM that regulates the ON/OFF of the MOSFETs S2, S4, and S6, DT_s indicates the duration when the MOSFETs S2, S4, and S6 are switched ON (i.e., $DT_s = dt_{a_{on}}$), $\Delta = 1 - D(i.e., \Delta T_s = dt_{a_{off}})$.

Because the current on the inductor cannot change abruptly, a rough estimate of the increase in current $dI_{a_{on}}$ through the inductor L_a equals to the decrease in current $dI_{a_{off}}$. (i.e., Eq. S18=Eq. S19). Thus, the average phase current $\overline{I_a}$ can be expressed as

$$\overline{I_a} = \frac{\left(E_b - E_a - \left((1 - D)\left(\overline{U_{uc}} + 2U_{FD}\right)\right)\right)}{2(R_a + R_s D)} \quad (S20)$$

Ultracapacitors only harvest energy when the average current $\overline{I_a} \geq 0$. To charge the ultracapacitor, we can obtain the following conditions

$$D \geq 1 - \frac{E_b - E_a}{\overline{U_{uc}} + 2U_{FD}} = D_b \quad (S21)$$

where D_b is the minimum PWM duty cycle that will charge the ultracapacitor.

Referring to Eq. S20, based on different cases 1-5, and the corresponding the average phase current $\overline{I_a}$ can be obtained. When MOSFETs are ON and OFF, the average charging current $\overline{I_{\text{charge}}}$ is equal to 0, and $\overline{I_a}$, respectively.

·*Case 1*: $D = 0$.

In this case, all MOSFETs are switched OFF. The circuit is simplified to six diodes in operation and the equivalent circuit is shown in Supplementary Fig. S7b. Therefore, the electrical forces of the circuit as distinct from Eq. S15 should be rediscussed. According to the expression for the induced electric forces, the difference between E_a and E_b can be expressed as

$$E_a - E_b = NBA\omega_{mo}sin(\omega_{mo}t) \quad (S22)$$

where *N* refers to the number of turns of the coil, *B* refers to the strength of the magnetic field, *A* represents the area enclosed by the single-turn coil, and ω_{mo} is the angular velocity of rotation of the rotor and *t* represents the time for the rotor to leave the initial neutral plane. While $E_a - E_b \leq U_{FD1} + U_{FD4} + U_{uc}$, the circuit does not form a closed loop, indicating no current flowing, i.e., $\overline{I_{charge}} = 0$ (Case 1.1). Otherwise, while $E_a - E_b > U_{FD1} + U_{FD4} + U_{uc}$, the charging current of

the ultracapacitor is $\overline{I_{charge}}$. Calculated from Eq. S16, $\overline{I_{charge}} = \frac{(E_b - E_a)e^{-t/RC}}{R}$ is obtained (Case 1.2), where R equals R_a plus R_b.

For cases 2, 3, and 4, it is roughly estimated that the circuit current does not change abruptly in one cycle, thus $\overline{I_a}$ is equal to $\overline{I_{charge}}$, when MOSFETs S2, S4 and S6 are OFF, as shown in Supplementary Fig. S7a(2).

·***Case 2***: $E_b - E_a \geq \overline{U_{uc}} + 2U_{FD}(i.e., D_b \leq 0)$ & $0 < D < 1$&S2, S4, S6 = OFF.

In this case, the regenerative supply voltage is always greater than the terminal voltage. According to Eq. S20, whatever the value of D is, the ultracapacitor is always able to be charged, i.e., $\overline{I_{charge}} = \frac{\left(E_b - E_a - \left((1-D)(\overline{U_{uc}} + 2U_{FD})\right)\right)}{2(R_a + R_s D)}$.

·***Case 3***: $E_b - E_a < \overline{U_{uc}} + 2U_{FD}(i.e., D_b > 0)$ & $0 < D < D_b$ & S2, S4, S6 = OFF

In this case, the MOSFETs *S2*, *S4*, and *S6* are switched ON for too short a time and the increase in current is low. When the MOSFETs are disconnected, there is not enough current to charge the ultracapacitor and the current is not continuously stable. Consequently, the current is less than $\frac{\left(E_b - E_a - \left((1-D)(\overline{U_{uc}} + 2U_{FD})\right)\right)}{2(R_a + R_s D)}$ and greater than 0.

·***Case 4***: $E_b - E_a < \overline{U_{uc}} + 2U_{FD}(i.e., D_b > 0)$ and $D_b < D < 1$&S2, S4, S6 = OFF.

In this case, the MOSFETs are switched on for a sufficiently long time. The increasing current enables the ultracapacitor to be continuously charged when the MOSFETs are OFF, indicating $\overline{I_{charge}} = \frac{\left(E_b - E_a - \left((1-D)(\overline{U_{uc}} + 2U_{FD})\right)\right)}{2(R_a + R_s D)}$.

For case 5, when MOSFETs S2, S4 and S6 are ON or D=1, $\overline{I_a}$ does not flow into the ultracapacitor, meaning that $\overline{I_{charge}}$ is equal to 0, as shown in Supplementary Fig. S7a(2) & Supplementary Fig. S7c.

·***Case 5***: $D = 1$ or S2, S4, S6 = ON

In this case, the ultracapacitor is not always connected to the closed-loop circuit and the phase current I_a in the circuit is $\frac{E_b - E_a}{2(R_a + R_s)}$. At this moment, all electrical energy is consumed in the form of heat, indicating $\overline{I_{charge}}$=0.

Ratio Calculation

The calculation equation of the ratio of regenerated power to consumed power is listed as follows. Regenerated power P_{reg} can be denoted as follows,

$$P_{reg} = \frac{\Delta Q_{cap}}{\Delta t_{cap}} = \frac{0.5CU_{cap2}^2 - 0.5CU_{cap1}^2}{t_2 - t_1} = \frac{(\int_0^{t_2} I_{charge}\, dt)^2 - (\int_0^{t_1} I_{charge}\, dt)^2}{2C(t_2 - t_1)} \quad (S23)$$

where U_{cap1} and U_{cap2} indicate the voltage of the ultracapacitor at the beginning time t_1 and the ending time t_2 of the experiment, respectively, and ΔQ_{cap} represents the energy collected by the ultracapacitor for the duration of Δt_{cap}.

Consumed power P_{con} can be expressed as follows,

$$P_{con} = \frac{Q_{con}}{\Delta t_{cap}} = U_{con} I_{con} \quad (S24)$$

where Q_{con} represents the electrical energy consumed by the isokinetic training robot, U_{con} and I_{con} represent the consumed voltage and current, respectively, during the operation of the isokinetic training robot. The ratio of regenerated power to consumed power η can be expressed as follows,

$$\eta = \frac{P_{reg}}{P_{con}} \times 100\% \quad (S25)$$

Comparison between Isokinetic Training and Self-Weight Training

The medical rationale for the effectiveness of isokinetic training lies in its ability to ensure that muscles generate maximal tension consistently throughout the full range of joint motion during the training process (*8-12*). To validate that isokinetic training leads to longer durations of maximal muscle activation compared to bodyweight exercises (e.g., squatting), an experiment was conducted to compare the duration of maximal muscle activation during isokinetic training and squatting. Muscle activation of the quadriceps (Fig. S8a) and hamstrings (Fig. S8b) was measured during both isokinetic training and squatting using EMG signals.

Both isokinetic training and squatting exhibit a trend of increasing muscle activity, reaching peak activation, and then decreasing. However, the duration of high muscle activation is significantly longer during isokinetic training compared to squatting. To quantitatively compare the duration of high muscle activation, the time during which the quadriceps and hamstrings experienced muscle activation greater than 80% of their maximum activation was calculated and compared. Figure S8a shows that the quadriceps experience 2.43 times longer high muscle activation during isokinetic training (51%) compared to squatting (21%). Similarly, Figure S8b demonstrates that the hamstrings experience 1.73 times longer high muscle activation during isokinetic training (38%) compared to squatting (22%). Both the quadriceps and hamstrings demonstrate that isokinetic training resulted in longer durations of high muscle activation compared to squatting, which may contribute to the superior effectiveness of isokinetic training over bodyweight exercises such as squatting.

Sample Size

The sample size was determined based on the following calculation:

$$N = 2 * \left[\left(Z_{\frac{\alpha}{2}} + Z_{\beta} \right) * \frac{S}{\delta} \right]^2 \quad (S26)$$

Given that $\alpha = 0.05$ and $\beta = 0.1$, the corresponding critical values are $Z_{\frac{\alpha}{2}} = 1.96$ and $Z_{\beta} = 1.28$. Based on the previous references, $S = 16.1$ and $\delta = 32.3$ (*33*). The calculation yielded a minimum required sample size of $N > 5.21$. To enhance the validity and rigor of the experiment, the study was conducted with 10 post-surgical subjects.

Supplementary Figure

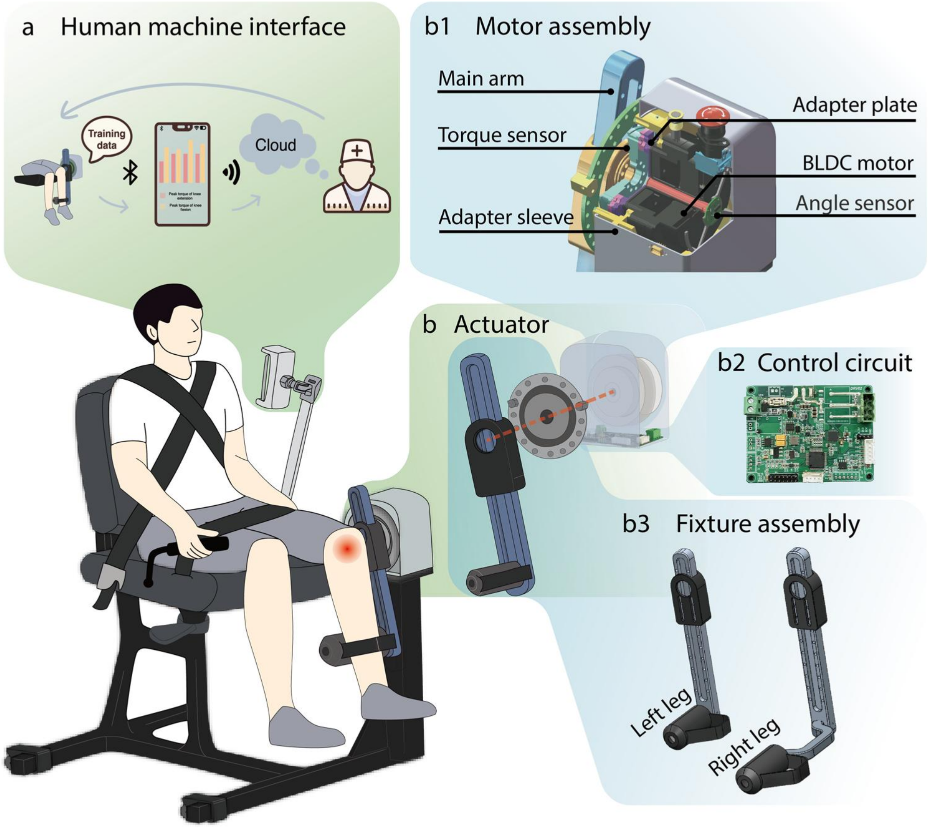

Fig. S1 | Detailed mechanical assembly. (a) Human-machine interface. Human-machine interface shows the real-time training data as feedback. Human-machine interface receives data from the control circuit via a Bluetooth model and sends data to the cloud. Doctors can diagnose remotely. **(b)** Actuator. (b1) Motor Assembly. Partially section view of the motor assembly. (b2) Control Circuit. (b3) Fixture assembly. Either of the left and right legs can use the isokinetic training robot, by changing the ankle fixture.

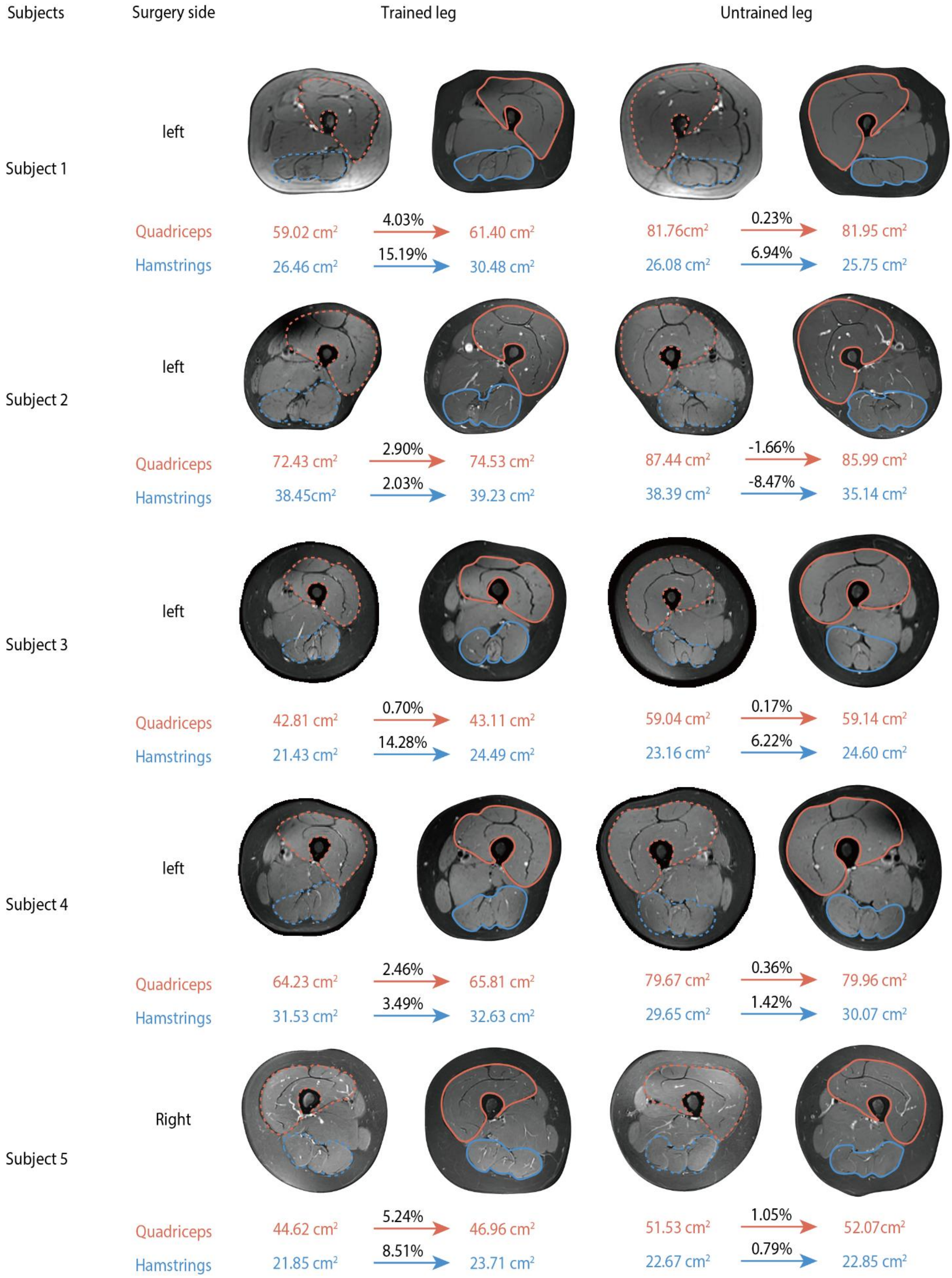

Fig. S2 | Magnetic resonance imaging (MRI) images of post-surgical subjects 1-5.

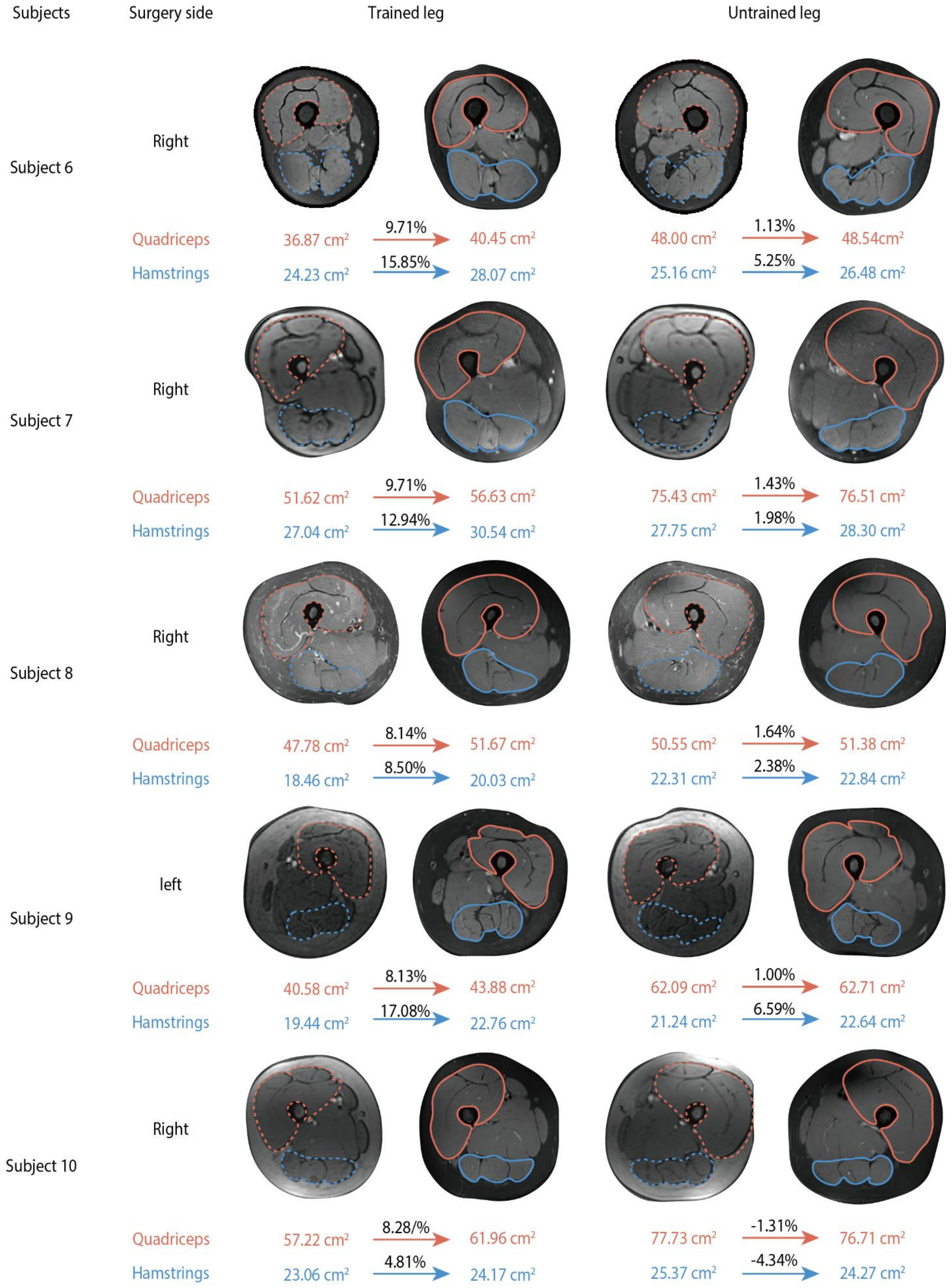

Fig. S3 | Magnetic resonance imaging (MRI) images of post-surgical subjects 6-10.

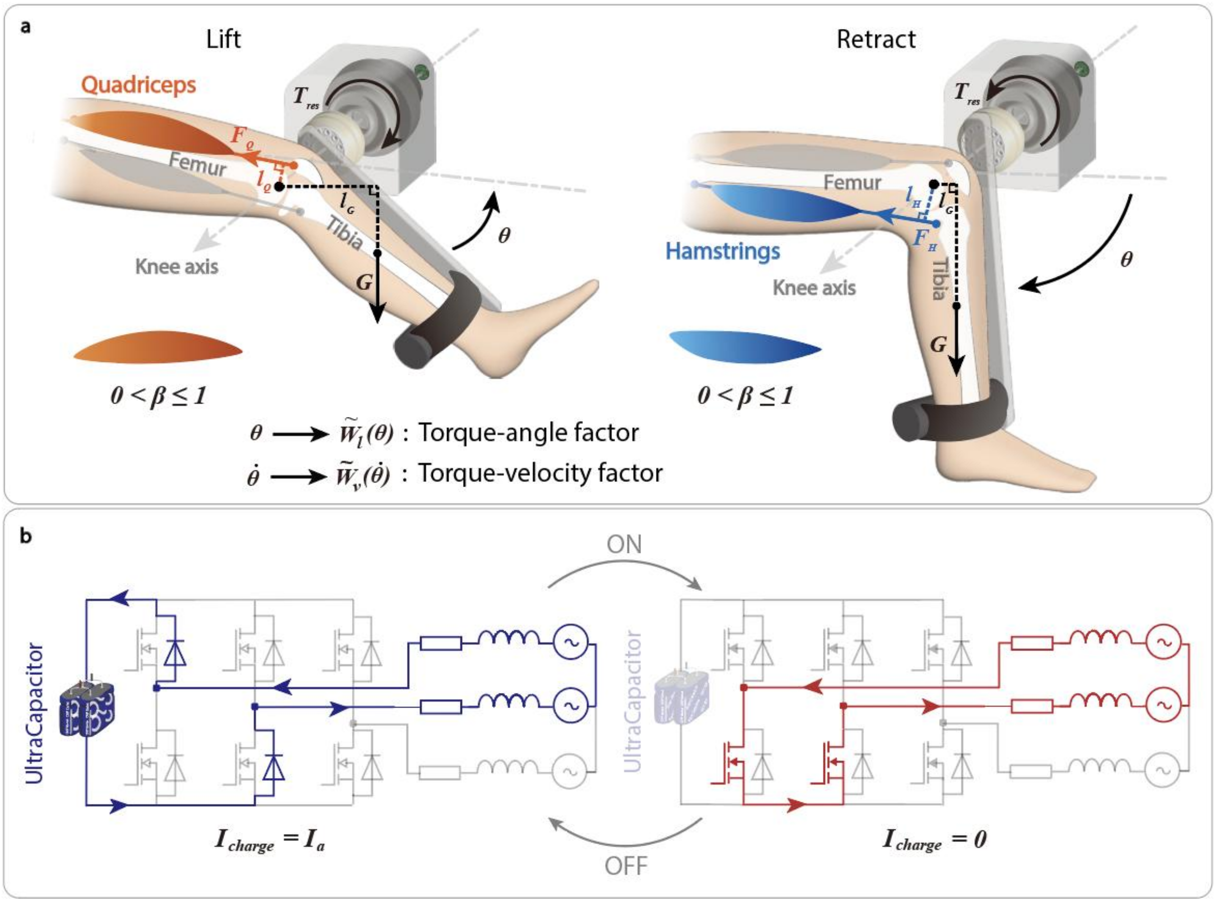

Fig. S4 | Energy regeneration during isokinetic training. (a) Lifting and retracting torque model. Quadriceps femoris contraction force F_Q dominates the lifting force, with the corresponding moment arm l_Q. Hamstring muscle contraction force F_H dominates the retracting force, with the corresponding moment arm l_H. β is a normalized muscle activation level. G is the gravitational force, and l_G is the corresponding moment arm. **(b)** Energy regeneration circuit. The low-side MOSFETs are switched ON/OFF. When the MOSFETs are OFF, the charging current I_{charge} equals the phase current I_a, and the current flows into the ultracapacitor. When the MOSFETs are ON, although the induced phase current exists, the charging current $I_{charge} = 0$.

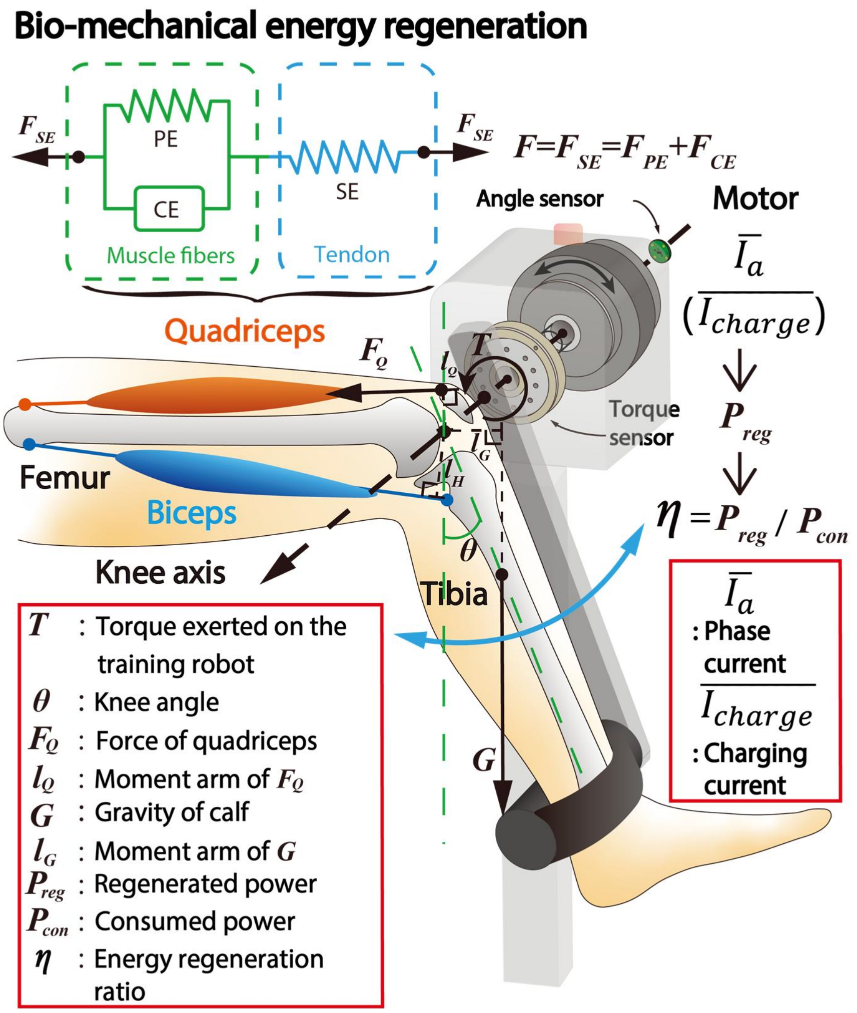

Fig. S5 | Model of bio-mechanical energy regeneration. Muscular-skeleton model of the human knee joint and three elements of a typical skeletal muscle according to Hill.

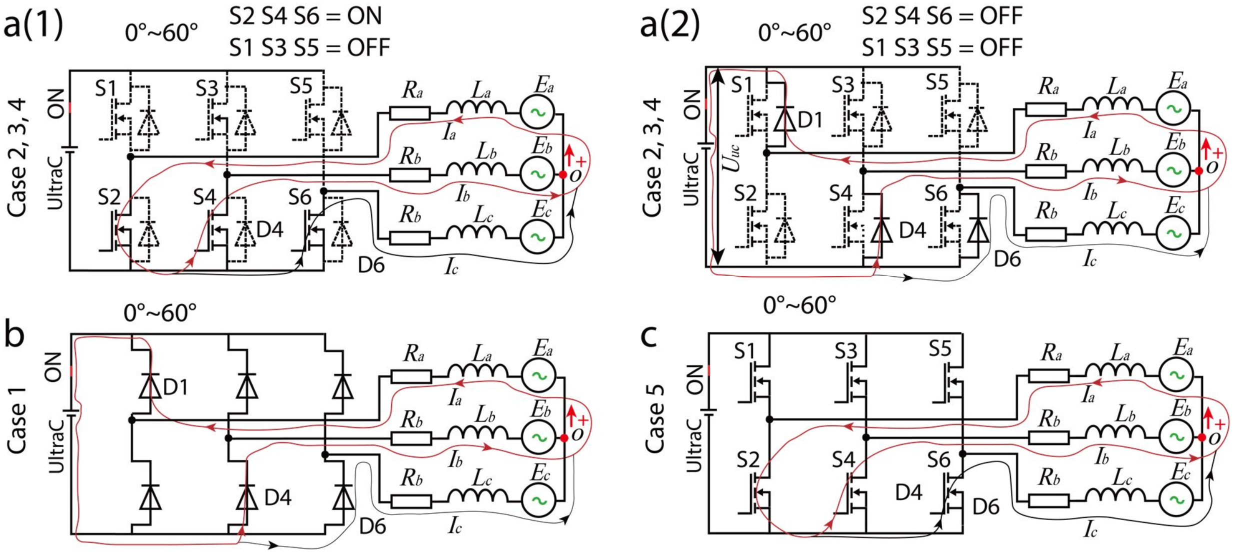

Fig. S6 | Equivalent braking/charging circuit in regeneration mode. (a) Cases 2, 3, 4. The MOSFETs S2, S4, and S6 are ON/OFF. **(b)** Case 1. The MOSFETs S2, S4, and S6 are always OFF during the entire cycle, showing the flow of the charging current when $max(E_a - E_b) = NBA\omega_{mo} > U_{FD1} + U_{FD4} + U_{uc}$. **(c)** Case 5. The MOSFETs S2, S4 and S6 are ON during the entire cycle. More details refer to Eq. S14-S22.

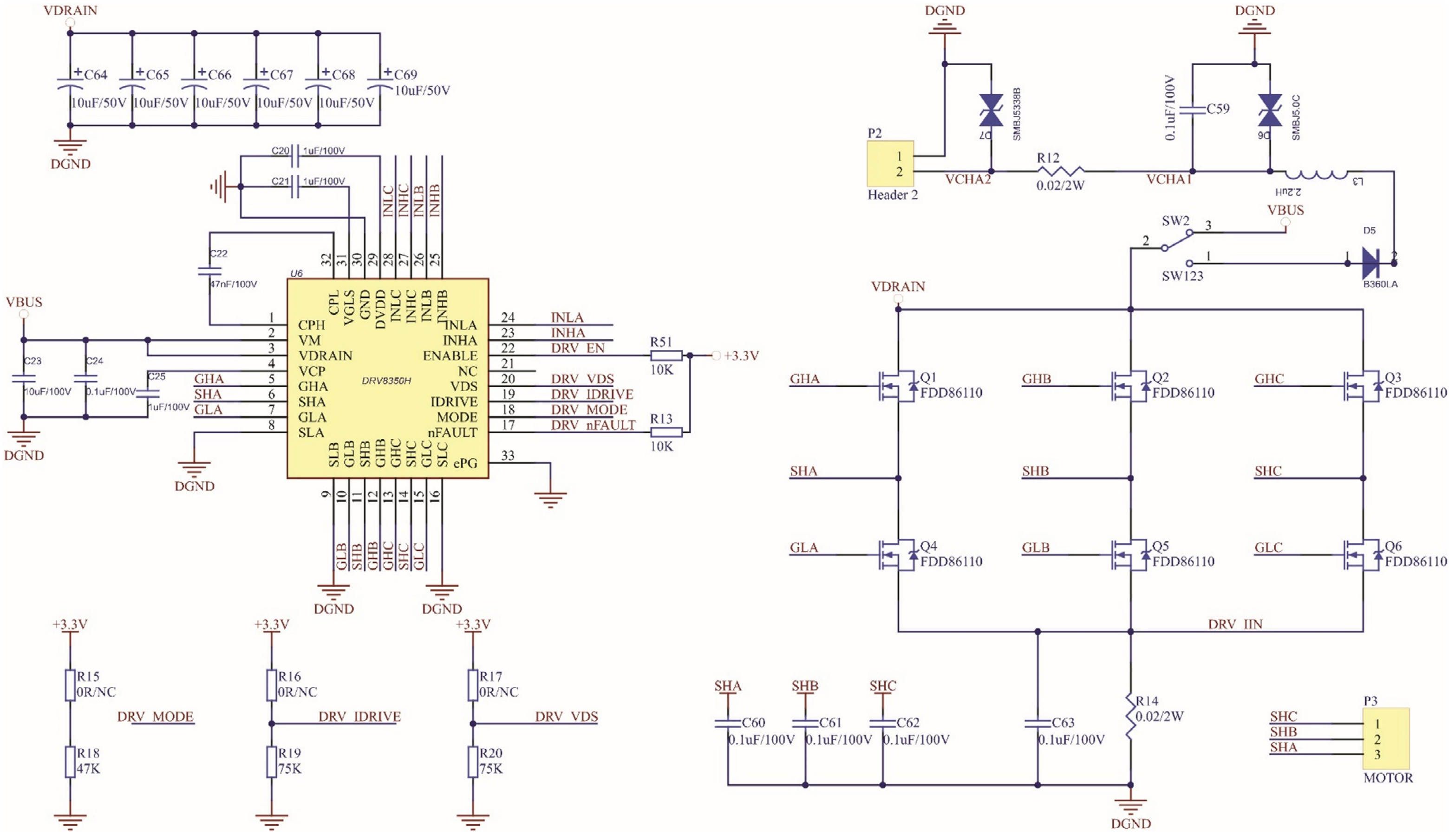

Fig. S7 | Motor driver circuit.

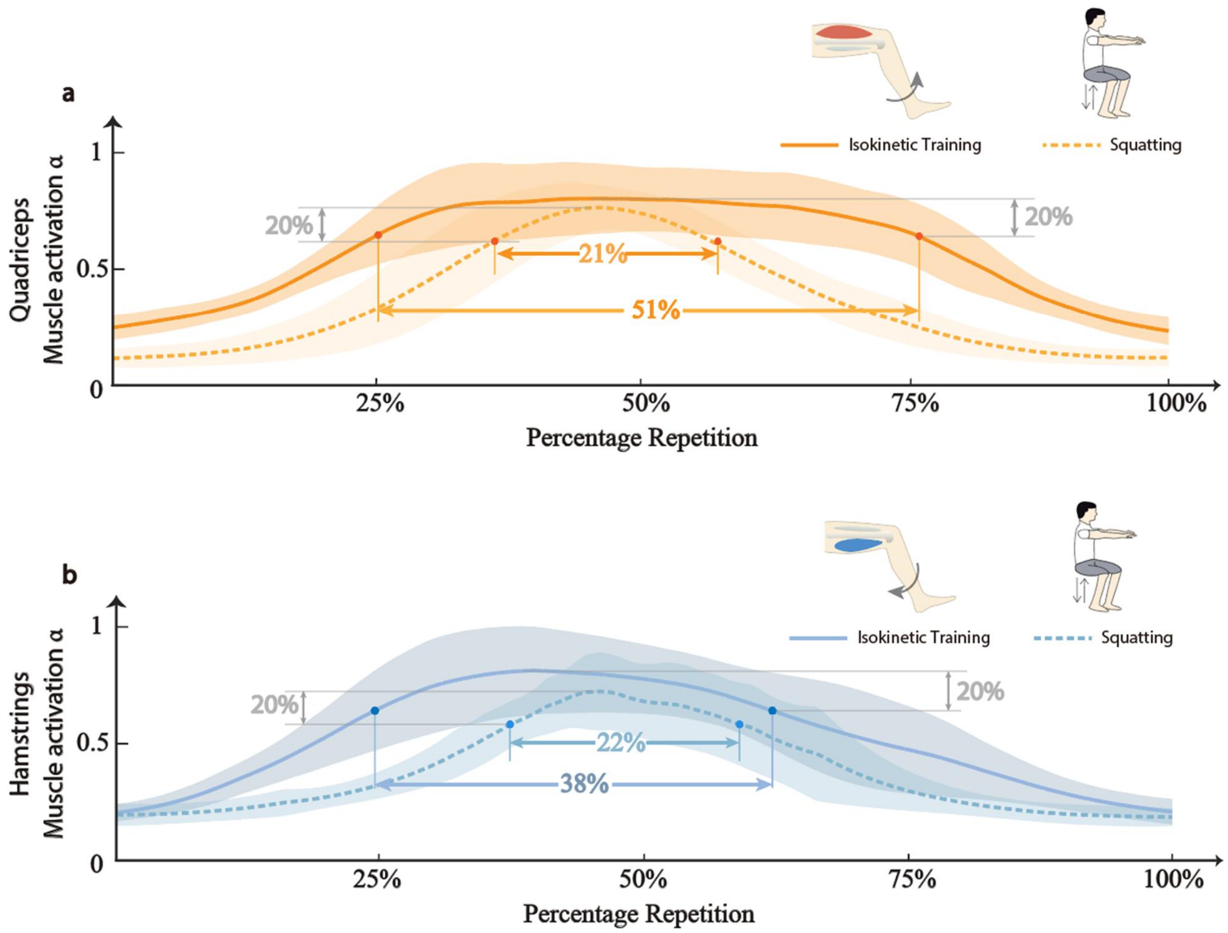

Fig. S8 | Muscle Activation Comparison between Isokinetic Training and Squatting. (a) Quadriceps muscle activation comparison. **(b)** Hamstring muscle activation comparison. The dashed line represents squatting, while the solid line represents isokinetic training. The orange line and dots represent quadriceps muscle activation, while the blue line and dots represent hamstring muscle activation. Data are presented as mean ± SD. Source data are provided as a Source Data file.

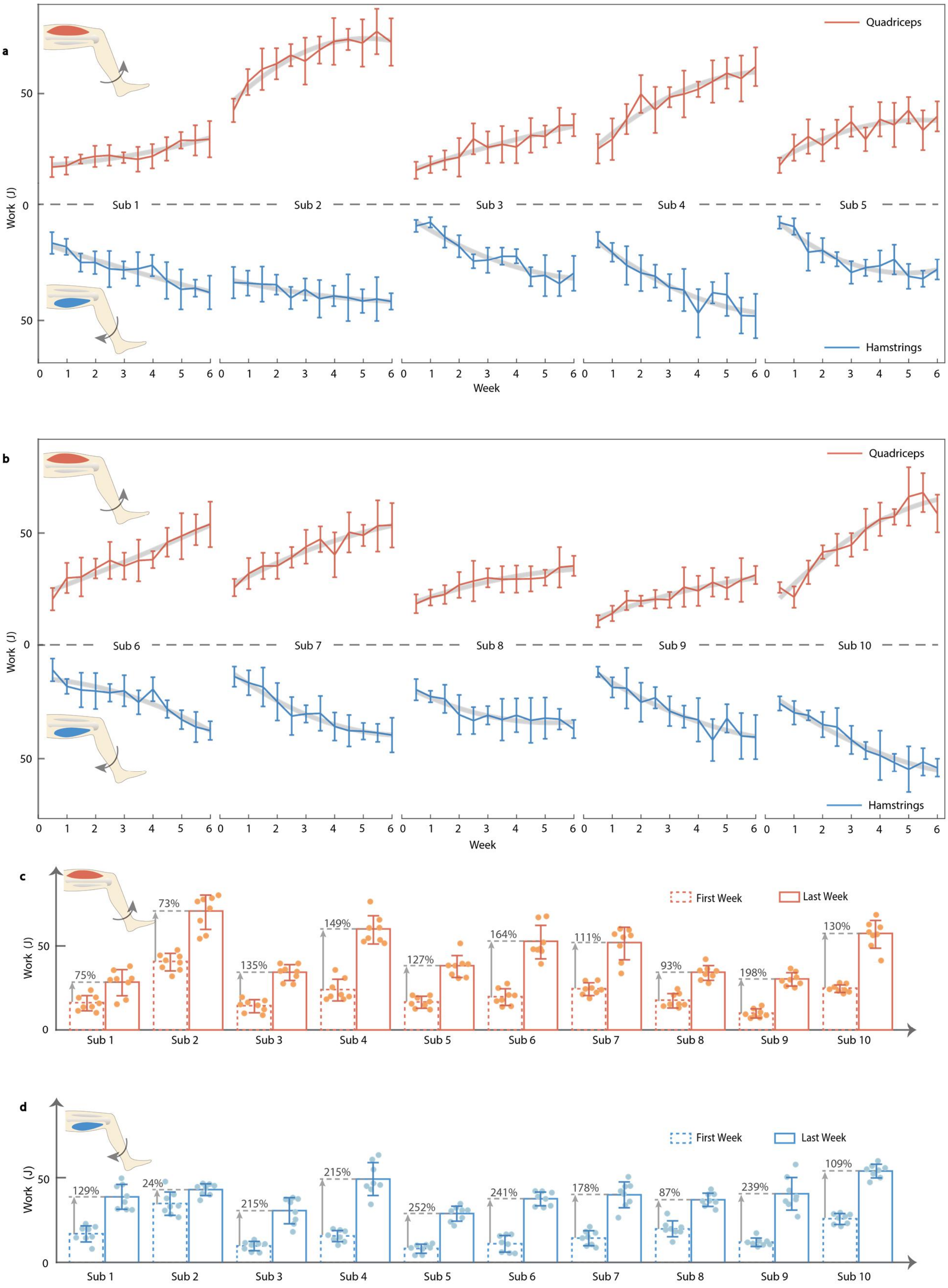

Fig. S9 | Evaluation of training intensity changes after a 6-week rehabilitation program. (a)-(b) Mean lifting and retracting work per repetition for 10 subjects over 6 weeks. **(a)** Subject 1-5. **(b)** Subject 6-10. **(c)-(d)** Progression of isokinetic training work from the first to the last week. **(c)**

Quadriceps work. **(d)** Hamstring work. Replicates: n=8. Source data are provided as a Source Data file.

Supplementary Tables

Table S1 | Comparison between existing hospital-based and our home-based rehabilitation robot

	Research	Power	Weight	Size	Human-machine Interface
**Commercial Product (Hospital-based)**	Biodex[19]	Immovable (230V, 3450W)	612 kg	2.92 (m^2)	22″ flat panel touchscreen
	Kineo[20]	Immovable (230V, 1600W)	345 kg	2.79 (m^2)	2×15.6” touch-screen displays
	CONTREX MJ[21]	Immovable (230V, 2300W)	350 kg	1.48 (m^2)	Display
	IsoMed2000[22]	Immovable (380V, 8000W)	650 kg	2.6 (m^2)	Display
**This Study (Home-based)**		Movable (Power-free)	52 kg	0.82 (m^2)	6.67″ Smart Phone

Table S2 | Post-surgical subject information. Detailed information of all post-surgical subjects (n=10).

Subject	Gender	Age (years)	Weight (kg)	Height (cm)	Calf length (cm)	Reason for surgery
1	Female	20-25	60-65	165-170	35-40	ACL
2	Male	40-45	80-85	175-180	40-45	ACL
3	Female	18-23	60-65	165-170	35-40	ACL
4	Male	20-25	85-90	175-180	40-45	ACL
5	Female	30-35	55-60	160-165	35-40	MCL
6	Female	45-50	55-60	165-170	35-40	ACL+MCL
7	Male	35-40	60-65	165-170	40-45	ACL+MCL
8	Female	30-35	50-55	160-165	35-40	ACL+MCL
9	Female	35-40	65-70	165-170	35-40	PD
10	Male	20-25	75-80	170-175	40-45	PD
**M(SD)**	**All**	31.7(9.7)	67.4(12.7)	167.4(4.8)	39.3(1.3)	

Notes: ACL: Anterior Cruciate Ligament, MCL: Medial Collateral Ligament, PD: Patellar Dislocation.

Table S3 | Subject information. Detailed information of all healthy subjects (n=10).

Subject	Gender	Age (years)	Weight (kg)	Height (cm)	Calf length (cm)
1	Female	20-25	40-45	155-160	35-40
2	Female	20-25	75-80	155-160	35-40
3	Female	20-25	55-60	165-170	35-40
4	Female	20-25	50-55	160-165	35-40
5	Male	20-25	65-70	175-180	40-45
6	Male	20-25	75-80	175-180	35-40
7	Male	20-25	75-80	180-185	40-45
8	Male	20-25	60-65	170-175	35-40
9	Female	25-30	60-65	165-170	40-45
10	Male	20-25	90-95	180-185	45-50
**M(SD)**	**All**	22.8(2.7)	65.8(15.9)	170.2(9.8)	39.1(3.4)

Table S4 | Power-free validation. Regeneration power and consumer power of healthy subjects (n=10) and post-surgical subjects (n=10).

		subject 1	subject 2	subject 3	subject 4	subject 5	subject 6	subject 7	subject 8	subject 9	subject 10
**Healthy Subjects**	**Regeneration power (W)**	1.62±0.18	1.97±0.50	2.21±0.14	2.25±0.29	2.35±0.22	2.42±0.03	2.62±0.21	2.85±0.25	3.69±0.16	4.66±0.28
	**Consumed power (W)**	1.24±0.03	1.27±0.04	1.28±0.04	1.27±0.03	1.25±0.04	1.27±0.03	1.24±0.04	1.26±0.04	1.24±0.02	2.25±0.04
	**Ratio (%)**	130%	155%	173%	177%	188%	191%	211%	227%	299%	373%
**Post-surgical Subjects**	**Regeneration power (W)**	2.20±0.61	2.61±0.87	1.76±0.66	2.67±1.03	1.81±0.44	2.52±0.50	2.29±0.96	2.72±0.65	1.50±0.50	2.38±1.02
	**Consumed power (W)**	1.24±0.01	1.20±0.02	1.17±0.03	1.19±0.02	1.18±0.02	1.20±0.02	1.21±0.01	1.25±0.01	1.16±0.03	1.27±0.03
	**Ratio (%)**	178%	217%	150%	224%	154%	210%	190%	218%	130%	187%

Table S5 | Tight Cross-sectional Area. Tight cross-sectional area of all post-surgical subjects (n=10) before and after 6-week training. BT: before training, AT: after training, Qua: quadriceps, Ham: hamstrings.

		subject 1	subject 2	subject 3	subject 4	subject 5	subject 6	subject 7	subject 8	subject 9	subject 10	mean±std
Trained leg	**Qua BT (cm^2)**	59.02	72.43	42.81	64.23	44.62	36.87	51.62	47.78	40.58	57.22	51.72±11.35
	**Qua AT (cm^2)**	61.4	74.53	43.11	65.81	46.96	40.45	56.63	51.67	43.88	61.96	54.64±11.26
	**Ham BT (cm^2)**	26.46	38.45	21.43	31.53	21.85	24.23	27.04	18.46	19.44	23.06	25.20±6.06
	**Ham AT (cm^2)**	30.48	39.23	24.49	32.63	23.71	28.07	30.54	20.03	22.76	24.17	27.61±5.73
	**Qua Increment (cm^2)**	2.38	2.10	0.30	1.58	2.34	3.58	5.01	3.89	3.30	4.74	2.92±1.46
	**Qua Increment (%)**	4.03%	2.90%	0.70%	2.46%	5.24%	9.71%	9.71%	8.14%	8.13%	8.28%	5.93%±3.27%
	**Ham Increment (cm^2)**	4.02	0.78	3.06	1.1	1.86	3.84	3.5	1.57	3.32	1.11	2.42±1.25
	**Ham Increment (%)**	15.19%	2.03%	14.28%	3.49%	8.51%	15.85%	12.94%	8.50%	17.08%	4.81%	10.27%±5.53%
Untrained leg	**Qua BT (cm^2)**	81.76	87.44	59.04	79.67	51.53	48	75.43	50.55	62.09	77.73	67.32±14.68
	**Qua AT (cm^2)**	81.95	85.99	59.14	79.96	52.07	48.54	76.51	51.38	62.71	76.71	67.50±14.23
	**Ham BT (cm^2)**	24.08	38.39	23.16	29.65	22.67	25s.16	27.75	22.31	21.24	25.37	25.98±5.06
	**Ham AT (cm^2)**	25.75	35.14	24.6	30.07	22.85	26.48	28.3	22.84	22.64	24.27	26.29±3.96
	**Qua Increment (cm^2)**	0.19	-1.45	0.1	0.29	0.54	0.54	1.08	0.83	0.62	-1.02	0.17±0.80
	**Qua Increment (%)**	0.23%	-1.66%	0.17%	0.36%	1.05%	1.13%	1.43%	1.64%	1.00%	-1.31%	0.40%±1.11%
	**Ham Increment (cm^2)**	1.67	-3.25	1.44	0.42	0.18	1.32	0.55	0.53	1.4	-1.1	0.32±1.50
	**Ham Increment (%)**	6.94%	-8.47%	6.22%	1.42%	0.79%	5.25%	1.98%	2.38%	6.59%	-4.34%	1.88%±5.00%

Table S6 | Maximum Lifting Torque. Maximum lifting torque of trained leg for all post-surgical subjects (mean±std, Nm).

Week	subject 1	subject 2	subject 3	subject 4	subject 5	subject 6	subject 7	subject 8	subject 9	subject 10
1	26.43±1.66	46.39±0.65	24.30±3.71	37.09±1.39	20.74±1.06	32.46±0.54	34.60±2.66	29.17±1.47	14.37±3.32	40.17±1.16
	25.69±1.31	60.16±3.67	29.15±1.44	39.50±1.03	30.67±0.99	41.67±1.81	43.04±0.49	29.34±1.85	19.82±1.76	33.18±0.69
2	27.83±2.45	66.51±3.98	30.72±1.53	48.61±1.98	35.60±1.08	44.90±1.44	49.55±1.09	31.90±0.43	26.81±2.63	46.19±1.35
	29.41±2.13	69.21±1.82	29.49±2.05	59.38±0.54	30.80±1.56	45.24±0.31	46.30±1.40	33.69±0.12	27.12±1.93	59.35±1.18
3	29.23±1.73	73.49±0.86	36.81±1.35	51.61±1.39	34.55±1.29	46.51±1.95	48.48±0.72	31.75±2.89	25.86±1.07	60.14±1.74
	28.76±2.43	70.53±2.31	34.25±1.40	56.32±2.37	40.67±1.33	52.24±1.04	51.90±2.19	34.68±1.48	22.64±1.27	62.15±1.68
4	27.49±2.77	76.00±1.32	37.93±0.78	56.65±1.64	31.94±3.52	50.65±4.60	55.46±1.69	35.88±1.19	31.30±2.95	63.50±2.20
	30.07±4.61	80.26±1.54	36.65±3.22	58.89±1.01	41.86±2.11	53.02±4.03	46.66±2.83	34.67±2.84	28.93±4.43	71.43±1.59
5	30.04±1.00	81.27±1.31	38.94±0.87	62.24±1.18	39.06±4.13	57.54±1.86	55.29±2.34	34.76±1.11	31.70±4.00	67.40±0.62
	32.72±1.64	79.48±1.84	41.11±3.06	70.28±1.19	46.33±1.44	61.70±0.85	53.70±0.87	33.89±1.98	29.16±3.94	74.25±1.07
6	31.71±1.14	85.18±2.14	38.79±1.34	62.97±0.87	36.43±1.86	57.02±0.97	58.46±0.97	38.23±0.84	33.19±2.11	78.94±1.89
	32.31±1.95	79.97±0.69	38.92±3.04	67.96±3.69	43.29±1.67	59.66±1.07	58.80±0.83	38.93±0.78	34.43±2.82	64.94±3.85

Table S7 | Maximum Retracting Torque. Maximum retracting torque of trained leg for all post-surgical subjects (mean±std, Nm). A negative value indicates torque generated in the leg retracting direction, which is the opposite of the leg lifting direction.

Week	subject 1	subject 2	subject 3	subject 4	subject 5	subject 6	subject 7	subject 8	subject 9	subject 10
**1**	-27.27±1.13	-39.50±1.47	-16.11±0.74	-23.99±0.80	-10.09±1.01	-17.90±1.39	-20.12±0.36	-32.30±1.24	-16.81±1.23	-41.45±0.94
	-28.22±1.88	-39.55±3.04	-13.87±1.07	-29.52±0.97	-12.35±1.13	-25.97±1.78	-23.79±0.83	-32.42±2.16	-27.29±1.76	-47.70±1.45
**2**	-35.74±1.45	-40.03±2.51	-23.47±1.36	-35.31±0.66	-25.53±1.58	-29.96±1.51	-26.95±1.47	-34.63±0.82	-26.60±1.43	-45.24±1.31
	-35.71±1.56	-40.29±2.30	-26.99±2.08	-37.40±1.18	-24.51±2.33	-27.42±0.78	-26.28±1.47	-39.64±0.36	-35.85±1.52	-51.34±1.05
**3**	-38.57±1.38	-46.88±1.15	-32.74±1.58	-39.77±1.85	-27.50±1.99	-26.46±2.39	-29.72±0.78	-38.03±2.10	-30.37±2.69	-52.31±1.30
	-40.45±1.30	-42.67±1.07	-34.42±2.32	-44.19±2.60	-34.13±1.11	-30.52±1.82	-35.92±0.79	-36.81±1.42	-33.75±2.15	-59.49±1.48
**4**	-39.36±2.88	-47.29±1.12	-33.63±0.51	-44.48±0.64	-31.77±3.31	-34.59±3.46	-32.91±0.60	-41.33±1.91	-39.27±2.91	-58.32±3.69
	-38.21±2.54	-45.88±1.91	-33.99±2.79	-56.27±0.87	-30.85±1.26	-27.67±3.11	-39.40±0.49	-37.48±2.05	-40.56±1.63	-63.21±1.63
**5**	-41.33±1.72	-46.74±0.57	-41.77±1.59	-45.28±2.51	-27.66±1.74	-36.06±1.64	-42.99±1.49	-40.09±1.76	-48.63±1.20	-62.28±2.03
	-43.52±1.20	-48.42±1.52	-43.99±1.91	-48.91±1.46	-36.07±2.12	-42.47±2.30	-43.22±0.62	-37.30±2.35	-38.27±3.92	-62.53±1.55
**6**	-41.62±1.43	-47.43±1.75	-39.71±1.77	-56.13±0.88	-37.24±3.10	-40.70±0.61	-44.03±0.99	-36.92±3.13	-47.03±1.40	-60.92±1.48
	-43.75±1.95	-48.59±0.76	-34.55±2.89	-55.61±2.12	-32.59±4.50	-42.47±0.59	-45.11±0.66	-41.82±1.94	-45.80±0.98	-61.00±3.28

Table S8 | Cost accounting

Gear Motor (LSG142-32-50)	Energy Regeneration Circuit	Torque Sensor (JH-NJLF)	GMR-Based Angle Sensor (TLE5012B)	Rack Machining	Total
$650.00	$60.00	$40.00	$10.00	$240.00	**$1,000.00**

Table S9 | Lifting Work. Mean lifting work of trained leg for all post-surgical subjects in one repetition (mean±std, J).

Week	subject 1	subject 2	subject 3	subject 4	subject 5	subject 6	subject 7	subject 8	subject 9	subject 10
**1**	16.41±4.41	40.92±5.14	14.67±3.74	24.22±6.43	16.90±3.35	20.01±4.91	24.74±3.78	17.90±4.11	10.25±2.63	25.05±2.33
	16.92±3.80	53.36±5.69	17.15±3.67	28.46±9.12	24.68±5.77	29.32±6.62	30.89±6.80	20.65±3.48	13.66±3.38	20.83±4.67
**2**	19.93±2.01	59.00±9.12	19.16±4.23	37.28±6.60	29.55±7.55	29.77±8.52	34.24±5.75	21.96±4.09	19.31±4.20	31.72±5.15
	21.10±4.65	61.40±6.82	20.38±8.45	48.29±8.29	25.69±6.98	33.48±4.34	34.21±5.67	26.17±5.66	19.16±2.29	40.59±2.95
**3**	21.55±4.54	65.19±4.87	28.48±6.78	41.32±10.12	30.64±6.27	37.04±8.11	38.00±4.52	27.87±8.86	19.90±4.05	41.64±7.00
	20.56±2.34	62.56±10.29	24.77±6.97	46.91±4.41	36.08±6.58	34.56±5.88	42.64±7.42	29.28±4.15	19.62±3.45	43.73±5.18
**4**	19.84±5.45	67.42±5.94	26.05±8.03	48.29±10.26	28.33±5.16	36.99±9.14	45.96±5.62	28.62±5.77	25.24±8.67	50.66±9.11
	21.16±5.33	71.20±10.26	24.91±7.12	50.37±3.36	37.13±7.53	37.38±3.72	39.12±9.86	28.73±5.97	23.57±6.60	55.08±7.40
**5**	24.39±5.15	72.09±4.65	30.02±7.59	53.76±9.29	34.65±9.72	45.01±6.33	49.04±8.88	28.85±5.53	27.34±6.28	56.14±3.49
	28.17±3.62	70.50±10.37	29.56±4.78	57.44±6.77	41.10±5.80	47.75±10.19	47.63±4.65	29.37±3.46	24.70±4.80	65.07±12.92
**6**	28.13±6.63	75.56±9.96	34.41±7.70	55.10±10.08	32.31±8.73	50.58±6.84	51.85±11.33	33.91±8.85	28.34±9.60	66.86±8.53
	28.66±7.97	70.94±10.40	34.52±4.86	60.28±8.47	38.40±6.60	52.92±10.08	52.16±9.84	34.54±4.40	30.54±3.97	57.61±8.34

Table S10 | Retracting Work. Mean retracting work of trained leg for all post-surgical subjects in one repetition (mean±std, J).

Week	subject 1	subject 2	subject 3	subject 4	subject 5	subject 6	subject 7	subject 8	subject 9	subject 10
**1**	16.93±4.78	34.84±6.88	9.73±2.66	15.66±3.41	8.22±2.69	11.04±4.98	14.38±4.44	19.82±4.66	12.00±2.45	25.85±3.13
	18.59±3.53	35.09±5.49	8.16±2.25	21.27±4.22	9.94±3.61	18.27±3.26	17.07±5.46	22.81±2.52	18.82±4.43	29.95±5.17
**2**	25.60±4.08	35.51±7.72	14.64±4.65	27.08±8.53	21.19±8.31	19.86±7.59	18.92±8.54	23.84±6.18	19.17±8.91	31.08±2.19
	25.63±5.07	35.74±4.39	18.65±4.82	30.42±7.98	20.45±5.42	20.29±7.90	25.40±8.24	30.79±8.67	25.33±8.57	35.12±5.56
**3**	28.44±8.04	41.58±4.86	25.34±3.13	31.84±5.04	24.39±3.22	21.07±3.88	31.68±8.33	33.38±5.90	23.37±4.68	36.22±8.36
	28.92±3.78	37.85±4.83	24.90±5.62	36.81±5.85	30.28±4.95	20.19±6.82	30.74±3.81	31.07±4.04	29.24±6.53	41.86±6.21
**4**	28.42±7.48	41.95±8.24	23.10±3.85	37.92±9.27	28.18±3.64	25.26±5.26	30.50±7.64	32.97±9.16	31.66±4.86	46.53±3.76
	26.89±4.76	40.70±4.49	23.11±3.19	48.13±10.59	27.37±6.89	19.51±5.26	36.27±6.16	31.05±7.41	33.04±9.55	48.74±10.93
**5**	33.55±8.06	41.46±10.26	32.21±4.17	39.11±4.70	24.54±6.60	28.21±3.86	38.13±6.92	33.27±9.88	41.93±9.19	51.87±3.88
	37.47±8.76	42.95±5.18	31.63±9.11	39.98±9.32	32.00±5.46	32.86±4.14	38.34±3.64	32.32±9.07	32.41±6.23	54.80±10.10
**6**	36.92±3.74	42.07±9.63	35.23±5.47	49.11±7.87	33.03±3.55	36.11±6.64	39.06±3.97	32.75±4.53	40.16±10.06	51.60±6.08
	38.80±7.37	43.10±3.58	30.64±7.75	49.33±9.74	28.91±4.44	37.67±4.09	40.01±7.61	37.10±4.00	40.62±9.68	54.11±4.00

46.Hill, A. V., The heat of shortening and the dynamic constants of muscle. *Proc. R. Soc. Lond., B, Biol. Sci*. **126**, 136-195 (1938).

47.Wang, X., Tao, X. & So, R. C. H. A bio-mechanical model for elbow isokinetic and isotonic flexions. *Sci. Rep.* **7**, 1-10 (2017).

48.Brown, I. E., Liinamaa, T. L. & Loeb, G. E. Relationships between range of motion, lo, and passive force in five strap-like muscles of the feline hind limb. *J. Morphol.* **230**, 69-77 (1996).

49.Wang, X. et al. Monitoring elbow isometric contraction by novel wearable fabric sensing device. *Smart Mater. Struct.* **25**, 125022 (2016).

50.Guo, J. et al. Continuous monitoring of electromyography (EMG), mechanomyography (MMG), sonomyography (SMG) and torque output during ramp and step isometric contractions. *Med. Eng. Phys.* **32**, 1032-1042 (2010).

51.Shi, J. et al. Continuous monitoring of sonomyography, electromyography and torque generated by normal upper arm muscles during isometric contraction: sonomyography assessment for arm muscles. *IEEE Trans. Biomed. Eng.* **55**, 1191-1198 (2008).
