# Supplementary material for: Power-free knee rehabilitation robot for home-based isokinetic training
Source: Nat Commun. 2025 Mar 8;16:2347. doi: 10.1038/s41467-025-57578-z (PMC11890594; doi:10.1038/s41467-025-57578-z)
Supplement: Supplementary file 2 — Description of Additional Supplementary Files [file 41467_2025_57578_MOESM2_ESM.docx]

**Description of Additional Supplementary Files**

**Supplementary Movie 1:** Overview of power-free and highly integrated isokinetic robot.

**Supplementary Movie 2:** A clinical experiment on a post-surgical subject.

**Supplementary Movie 3:** Post-surgical subject information.
