# Supplementary material for: Power-free knee rehabilitation robot for home-based isokinetic training
Source: Nat Commun. 2025 Mar 8;16:2347. doi: 10.1038/s41467-025-57578-z (PMC11890594; doi:10.1038/s41467-025-57578-z)
Supplement: Supplementary file 6 — Reporting Summary [file 41467_2025_57578_MOESM6_ESM.pdf]

nature portfolio

Corresponding author(s): Yanggang Feng, Wuxiang Zhang, Liu Wang, Xilun Ding

Last updated by author(s): Jan 26, 2025

Reporting Summary

Nature Portfolio wishes to improve the reproducibility of the work that we publish. This form provides structure for consistency and transparency in reporting. For further information on Nature Portfolio policies, see our Editorial Policies and the Editorial Policy Checklist.

Statistics

For all statistical analyses, confirm that the following items are present in the figure legend, table legend, main text, or Methods section.

n/a	Confirmed	
☐	☒	The exact sample size (*n*) for each experimental group/condition, given as a discrete number and unit of measurement
☐	☒	A statement on whether measurements were taken from distinct samples or whether the same sample was measured repeatedly
☐	☒	The statistical test(s) used AND whether they are one- or two-sided<br>*Only common tests should be described solely by name; describe more complex techniques in the Methods section.*
☒	☐	A description of all covariates tested
☐	☒	A description of any assumptions or corrections, such as tests of normality and adjustment for multiple comparisons
☐	☒	A full description of the statistical parameters including central tendency (e.g. means) or other basic estimates (e.g. regression coefficient) AND variation (e.g. standard deviation) or associated estimates of uncertainty (e.g. confidence intervals)
☐	☒	For null hypothesis testing, the test statistic (e.g. *F*, *t*, *r*) with confidence intervals, effect sizes, degrees of freedom and *P* value noted<br>*Give P values as exact values whenever suitable.*
☒	☐	For Bayesian analysis, information on the choice of priors and Markov chain Monte Carlo settings
☒	☐	For hierarchical and complex designs, identification of the appropriate level for tests and full reporting of outcomes
☒	☐	Estimates of effect sizes (e.g. Cohen's *d*, Pearson's *r*), indicating how they were calculated

Our web collection on statistics for biologists contains articles on many of the points above.

Software and code

Policy information about availability of computer code

Data collection	Data from the isokinetic training device and clinical data were collected using a smartphone software interface, a PC software interface and a Siemens Medical MRI device.
Data analysis	All data were analyzed using the SPSS 25.0 statistical package and Microsoft Excel.

For manuscripts utilizing custom algorithms or software that are central to the research but not yet described in published literature, software must be made available to editors and reviewers. We strongly encourage code deposition in a community repository (e.g. GitHub). See the Nature Portfolio guidelines for submitting code & software for further information.

Data

Policy information about availability of data

All manuscripts must include a data availability statement. This statement should provide the following information, where applicable:

- Accession codes, unique identifiers, or web links for publicly available datasets
- A description of any restrictions on data availability
- For clinical datasets or third party data, please ensure that the statement adheres to our policy

The data supporting the results of this study are available within the paper and its Supplementary Information. Additionally, source data is provided in a separate Microsoft Excel file.

Research involving human participants, their data, or biological material

Policy information about studies with human participants or human data. See also policy information about sex, gender (identity/presentation), and sexual orientation and race, ethnicity and racism.

Reporting on sex and gender	4 males and 6 females
Reporting on race, ethnicity, or other socially relevant groupings	Not involved
Population characteristics	1.Have done knee surgery before. 2.Age 18-75 years. 3. Self-reported reduction in tight muscle strength. 4. BMI<35
Recruitment	All participants provided informed consent and were recruited from within the research group.
Ethics oversight	Each subject was provided written informed consent, approved by the Biomedical Ethics Review Committee of West China Hospital, Sichuan University(No.20231559)

Note that full information on the approval of the study protocol must also be provided in the manuscript.

Field-specific reporting

Please select the one below that is the best fit for your research. If you are not sure, read the appropriate sections before making your selection.

[x] Life sciences [ ] Behavioural & social sciences [ ] Ecological, evolutionary & environmental sciences

For a reference copy of the document with all sections, see nature.com/documents/nr-reporting-summary-flat.pdf

Life sciences study design

All studies must disclose on these points even when the disclosure is negative.

Sample size	10
Data exclusions	No data was excluded.
Replication	The relability of the isokinetic training robot was verified by the 6-week rehabilitation process (12 sessions).
Randomization	Participants were randomly recruited, with 10 subjects selected from a pool of 300 post-surgical individuals.
Blinding	No blinding measures were intentionally implemented in this study.

Reporting for specific materials, systems and methods

We require information from authors about some types of materials, experimental systems and methods used in many studies. Here, indicate whether each material, system or method listed is relevant to your study. If you are not sure if a list item applies to your research, read the appropriate section before selecting a response.

Materials & experimental systems

n/a	Involved in the study	
x		Antibodies
x		Eukaryotic cell lines
x		Palaeontology and archaeology
x		Animals and other organisms
	x	Clinical data
x		Dual use research of concern
x		Plants

Methods

n/a	Involved in the study	
x		ChIP-seq
x		Flow cytometry
x		MRI-based neuroimaging

Clinical data

Policy information about clinical studies

All manuscripts should comply with the ICMJEguidelines for publication of clinical research and a completedCONSORT checklist must be included with all submissions.

Clinical trial registration	ChiCTR2300076715
Study protocol	Approved by the Biomedical Ethics Review Committee of West China Hospital, Sichuan University(No.20231559)
Data collection	Data collection was conducted in each training sessions, from the recruitment to the termination of the training sessions (6 weeks).
Outcomes	1.MRI 2.muscle isokinetic strength

Plants

Seed stocks	None
Novel plant genotypes	None
Authentication	None
